# Circulating biomarkers in pulmonary arterial hypertension: State-of-the-art review and future directions

**DOI:** 10.1016/j.jhlto.2024.100152

**Published:** 2024-08-23

**Authors:** Salaheldin Ahmed, Abdulla Ahmed, Göran Rådegran

**Affiliations:** a Department of Clinical Sciences Lund, The Section for Cardiology, Lund University, Lund, Sweden; b The Haemodynamic Lab, The Section for Heart Failure and Valvular Disease, VO Heart and Lung Medicine, Skåne University Hospital, Lund, Sweden; c Department of Education and Research, Helsingborg Hospital, Helsingborg, Sweden

**Keywords:** biochemical markers, pulmonary arterial hypertension, pulmonary hypertension, omics, high-throughput technologies, European Medicines Agency (EMA), Food and Drug Administration(FDA), pathology

## Abstract

Pulmonary arterial hypertension is a complex and heterogeneous condition, associated with a considerable diagnostic delay, diminished exercise capacity, and poor outcomes. In pulmonary arterial hypertension, biomarker research has become a subject of intense inquiry, and novel circulating biomarkers acknowledged in a multitude of mechanistic pathways are emerging. Beyond the widely used natriuretic peptides, novel biomarkers may provide deeper pathophysiological understanding, support clinical decision-making, and prompt the incorporation of precision medicine by enabling a more precise individual phenotyping. In this state-of-the-art review, the recent advances in circulating biomarkers in pulmonary arterial hypertension from a clinical perspective are discussed, with particular emphasis on the current state of knowledge, gaps in evidence, and future perspectives.

## Graphical Abstract

**Figure:**
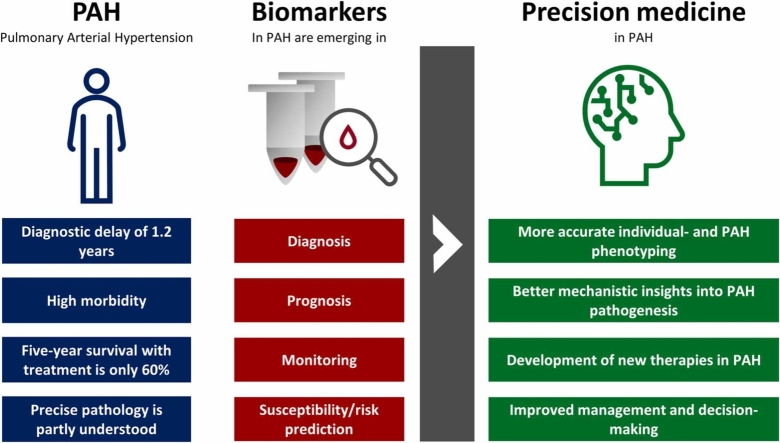

## Background

Pulmonary arterial hypertension (PAH) is a progressive angioproliferative vasculopathy affecting predominantly the small pulmonary arteries, involving all layers of the pulmonary vasculature.^1^, ^2^, ^3^ It is induced and perpetuated by an intricate interplay of genetic, environmental, structural, and/or functional perturbations, resulting, among other mechanisms, in deranged metabolic-, immunomodulatory- and inflammatory responses.^1^, ^4^ Collectively, these mechanisms promote endothelial dysfunction, excessive vasoconstriction, and loss of vascular compliance, as well as vascular remodeling, characterized by intimal proliferation and medial hyperplasia, in situ thrombosis, and formation of plexiform lesions.^1^, ^5^ The resultant rise in pulmonary vascular resistance integrated with the presence of precapillary pulmonary hypertension (PH), ultimately lead to impaired right-ventricular–pulmonary arterial coupling, right-ventricular failure, and premature death.^1^, ^2^

Despite the immense efforts and advances made in PAH management and treatment over the past decades, the prognosis of PAH remains poor.^2^, ^6^, ^7^, ^8^, ^9^ Furthermore, the pathophysiology of PAH is incompletely understood, which in part, may be explained by the heterogeneity and the complexity of the disease.^1^, ^4^ Currently available therapies lack sufficient hemodynamic efficacy and target primarily the excessive vasoconstrictive component.^1^ Also, no approved therapies have been demonstrated to cure or yet to clearly reverse the progression of the vascular disease in PAH, although promising early hemodynamic responses have been shown with Sotatercept.^5^, ^10^ Apart from imposing a significant societal burden in terms of cost, morbidity, and mortality, PAH is still accompanied by a substantial median diagnostic delay of 1.2 years (mean 2.03), compared to the median time of 1.27 years (mean 2.5) reported by the National Institutes of Health Primary Pulmonary Hypertension registry in the 1980s.^4^, ^11^, ^12^ These numbers correspond to the French registry and the Registry to Evaluate Early and Long-term Pulmonary Arterial Hypertension Disease Management (REVEAL), reporting a mean diagnostic delay of 2.25 and 2.84 years, respectively, between 2002 and 2007.^13^, ^14^ A recent multinational study conducted between 2019 and 2020 found that the diagnostic delay for PAH differed across regions, averaging from 0.83 to 2.0 years as reported by patients, and from 0.77 to 1.52 years as reported by physicians.^15^ These results may signify an overall positive trend toward an improved time to diagnosis, but also the need to further concurrent screening strategies to obtain a timely diagnosis and increase awareness of the disease.

An emerging tool across the wide spectrum of various conditions in both health and disease constitutes biomarkers.^16^, ^17^ Beyond the widely used natriuretic peptides in PAH, novel biomarkers may facilitate the incorporation of precision medicine by enabling more accurate disease characterization and individual phenotyping.^4^, ^16^, ^17^ Biomarkers may also improve disease management and support clinical decision-making, as well as offer more profound mechanistic insights into the disease pathogenesis, prompting the development of new therapies in PAH.^1^, ^4^, ^16^, ^18^

## Methods and results

### Search strategy

The searches were conducted in PubMed (Medline, PMC, NCBI Bookshelf) and Embase, and included the same entry terms outlined in section 6.2.5. “*Biochemical markers*,” in the 2022 European Society of Cardiology/European Respiratory Society (ESC/ERS) PH guidelines.^5^ The searches were done on September 15, 2023, and were coordinated with the Lund University Library. The search entry terms were based on the following keywords: *pulmonary arterial hypertension*, *vascular remodeling, biomarkers*, and *proteome*. For the full overview, see Appendix 1.

### Study selection and inclusion criteria

The selection of eligible studies was conducted by 2 independent reviewers (S.A. and A.A.) by applying prespecified inclusion and exclusion criteria (Table 1), as well as a consensus between the authors in case of uncertainty. Title, abstract, and full-text screening of the literature were conducted in Covidence—a systematic review software, Veritas Health Innovation, Melbourne, Australia; available at www.covidence.org.

**Table 1:** Eligibility Criteria Used for Selection of Circulating Biomarker Studies in Pulmonary Arterial Hypertension

Inclusion criteria	Exclusion criteria
Human studies (adults)	Pediatric studies
	Studies with results of pooled PH population (group 1 with 2-5)
	Animal studies
	In vivo/in vitro studies
	Low power in relation to the study design and purpose
Circulating biochemical markers of PAH	Noncirculating biochemical markers of PAH (i.e., MRI, echocardiography, other modalities)
Hemodynamic assessment/diagnosis supported by right heart catheterization	Pulmonary hypertension diagnosis based on echocardiography
Main outcome is clinically relevant, e.g., directly associated with PAH prognosis, diagnosis, treatment response, monitoring, or risk assessment	Main outcome is not PAH focused
	Gene-expression studies
Articles published from 2012 through September 2023	Articles published before 2012
	Reviews, guidelines, case reports, letters, expert statements, conference abstracts

Abbreviations: MRI, magnetic resonance imaging; PAH, pulmonary arterial hypertension; PH, pulmonary hypertension.

### Search strategy results

**Figure 1:**
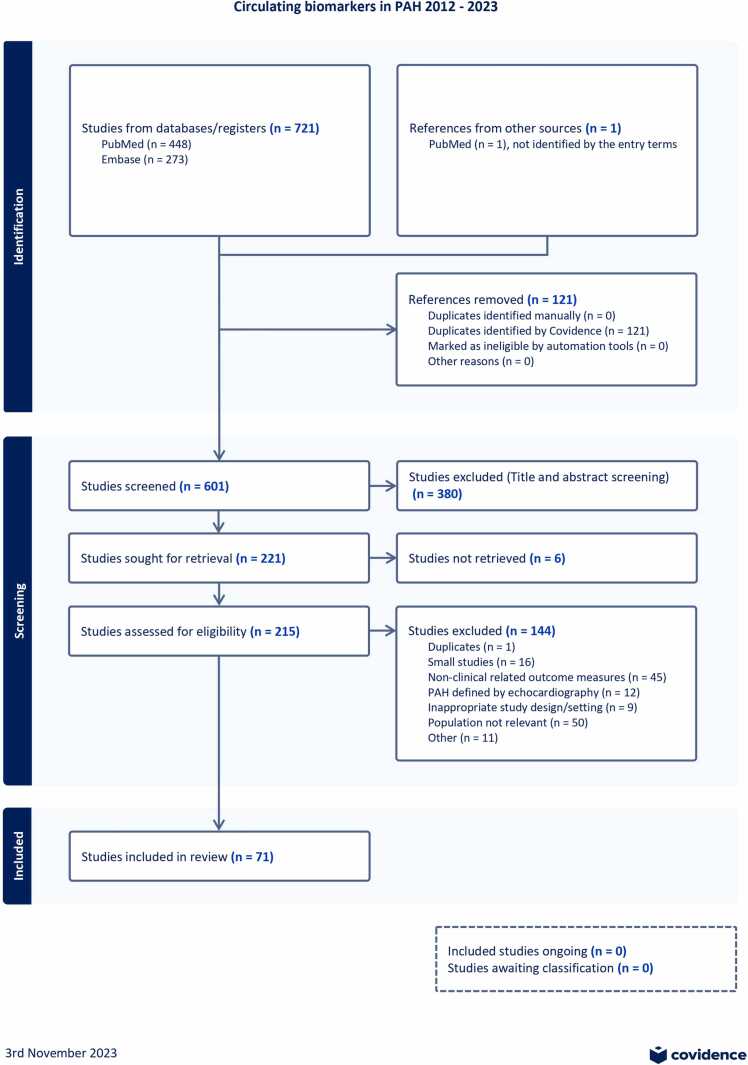
Preferred reporting items for systematic reviews and meta-analyses flow diagram displaying the study selection process. Eligibility criteria used during screening are defined in (Table 1). PAH, pulmonary arterial hypertension.

The comprehensive search identified 722 studies, of which 121 were duplicates. The title and abstract screening resulted in a total of 221 studies eligible for full-text reviewing. In the final synthesis, 71 studies were included. The article selection strategy is presented in Figure 1. An overview of the included studies with corresponding biomarker candidates and their potential clinical utility (class) is presented in Table 2.

**Table 2:** Overview of Included Circulating Biomarker Studies in Pulmonary Arterial Hypertension With Corresponding Biomarker Candidates and Their Potential Clinical Utility (Class)

Author	Biomarker candidate	Biospecimen	Class/clinical use	Population	AUCs	Validation
Alotaibi et al^19^	11-Testosterone, 17β estradiol, FAHFA, lignoceric acid, LTB_4_, nervonic acid, nitrooleate, novel eicosanoid, prostaglandin F2a	Plasma	Diagnosis, monitoring	IPAH, APAH-SSc	0.84-0.86	Externally validated
Simpson et al^20^	13 Metabolic profiles including (arginine, tryptophan, kynurenine pathways)	Plasma	Monitoring	IPAH, APAH-SSc	0.86	Not validated
Zhang et al^21^	15-F2t-isoprostane	Plasma	Prognosis, monitoring	IPAH	0.77	Not validated
Bouzina et al^22^	ACE2, adrenomedullin, renin	Plasma	Prognosis, monitoring	PAH (majority IPAH, APAH-SSc)	NA	Not validated
Guignabert et al^23^	Activin A, FSTL3	Serum	Prognosis	IPAH/HPAH	0.73-0.86	External validation
Ahmed et al^24^	ADAMTS13, vWF	Plasma	Diagnosis, prognosis, monitoring	PAH (majority IPAH, APAH-SSc)	0.78-0.94	Internally validated
Li et al^25^	ADMA, BNP, endothelin 1, galectin 3, uric acid	Plasma	Monitoring	APAH-CHD	NA	Not validated
Ahmed et al^26^	AMBP, glyoxalase I, LPL	Plasma	Diagnosis	PAH (majority IPAH, APAH-SSc)	0.79-0.87	Internally validated
Becker et al^27^	Anti-AT_1_R-antibodies, anti-ET_A_R antibodies	Serum	Susceptibility/risk prediction, prognosis, diagnosis	APAH-SSc, APAH-CTD	0.66-0.79	Not validated
Arvidsson et al^28^	Annexin A-1, CEACAM8, TRAIL	Plasma	Diagnosis, prognosis, monitoring	PAH (majority IPAH, APAH-SSc)	0.70^a^	Not validated
Rhodes et al^18^	Apolipoprotein E, CFD, CFH, erythropoietin, IGFBP-1, ST2, plasminogen, TIMP1, TIMP2	Plasma	Prognosis, monitoring	IPAH, HPAH	0.74,^b^ 0.83-0.93	Externally validated
Yogeswaran et al^29^	AST/ALT ratio, GGT, NLR	Blood	Prognosis, monitoring	IPAH	NA	Externally validated
Boucly et al^30^	ß-NGF, CXCL9, and TRAIL	Serum	Monitoring, prognosis	IPAH/HPAH, drug-induced PAH	~0.70-0.75^c^	Externally validated
Hartopo et al^31^	Bioactive adrenomedullin	Plasma	Prognosis	IPAH, APAH-ASD	NA	Not validated
Nikolic et al^32^	BMP-9	Plasma	Diagnosis, prognosis	PoPH, PAH (diverse groups)	0.83-0.99^d^	Externally validated
Frantz et al^33^	BNP	Plasma	Prognosis	PAH (diverse groups)	NA	Not validated
Heresi et al^34^	Cardiac troponin I	Plasma	Monitoring, prognosis	IPAH, APAH-CTD	NA	Not validated
Hoffmann-Vold et al^35^	CCL21	Serum	Susceptibility/risk prediction, monitoring, prognosis	APAH-SSc	NA	Externally validated
Brusca et al^36^	Cell-free DNA	Plasma	Prognosis, monitoring	PAH (diverse groups)	0.72-0.78	Partially externally validated
Sanges et al^37^	Chemerin	Serum	Monitoring	APAH-SSc	NA	Externally validated
Zhang et al^38^	circ_0068481 (circular RNA)	Serum	Diagnosis, prognosis, monitoring	IPAH	0.90-0.99	Not validated
Bauer et al^39^	Collagen IV, endostatin, IGFBP-2, IGFBP-7, MMP-2, neuropilin 1, NT-proBNP, RAGE	Serum	Diagnosis	APAH-SSc	0.74-0.87	Externally validated
Nickel et al^40^	Copeptin	Serum	Prognosis, monitoring	IPAH, APAH-CTD, APAH-CHD	0.77	Not validated
Kolditz et al^41^	Copeptin, MR-proADM, NT-proBNP	Plasma	Monitoring, prognosis	IPAH, APAH-CTD, PoPH	0.73-0.84	Not validated
Liu et al^42^	CTGF, NT-proBNP	Plasma	Monitoring, diagnosis	APAH-CHD	0.68-0.90	Not validated
Li et al^43^	CXCL1, CXCL8, CXCL10, CXCL12	Serum	Diagnosis, monitoring	IPAH	0.72-0.88	Not validated
Van Bon et al^44^	CXCL4	Plasma	Susceptibility/risk prediction, monitoring	APAH-SSc	NA	Externally validated
Simpson et al^45^	Endostatin	Serum	Prognosis, monitoring	PAH (majority IPAH and APAH-CTD)	0.73-0.79	Not validated
Damico et al^46^	Endostatin	Serum	Diagnosis, prognosis, monitoring	IPAH, APAH-CTD	0.68, 0.88^e^	Externally validated
Harbaum et al^47^	Endoglin, GDF-15, IGFBP-7, netrin-4, RNASE1, spondin-1, SVEP1, thrombospondin-2	Plasma	Prognosis	IPAH/HPAH	NA	Externally validated
Bouzina et al^48^	FGF-23, LOX1, NT-proBNP	Plasma	Monitoring	PAH (majority IPAH, APAH-SSc)	NA	Not validated
Rice et al^49^	FSTL3, midkine	Serum	Diagnosis	APAH-SSc	0.76-0.96	Externally validated
Scelsi et al^50^	Galectin 3	Plasma	Monitoring	IPAH, HPAH, APAH-SSc	NA	Not validated
Qian et al^51^	GDF-15, NT-proBNP	Serum	Prognosis, diagnosis	APAH-SLE	0.72-0.84	Not validated
Lu et al^52^	GGT, NT-proBNP	Serum	Prognosis, monitoring	IPAH	0.70, 0.77^f^	Not validated
Yang et al^53^	HDGF	Serum	Prognosis, diagnosis, monitoring	PAH (majority IPAH and APAH-CTD), PoPH	NA	Not validated but is an external validation of^54^
Yang et al^54^	HDGF	Serum	Prognosis, monitoring	IPAH, APAH-CTD, APAH-CHD	0.89	Externally validated
Al-Naamani et al^55^	HDL, NT-proBNP, thromboxane B_2_, total cholesterol, vWF	Plasma	Prognosis, monitoring	PAH (majority IPAH, APAH-CTD)	NA	Not validated
Harbaum et al^56^	HDL-4-Apo A-1, HDL-4-Apo A-2, HDL-4-phospholipids	Plasma	Prognosis	IPAH, HPAH	0.57-0.73	Internally validated
Belly et al^57^	Hemoglobin A1c (HbA1c)	Blood	Prognosis	IPAH, APAH-CTD, APAH-CHD, PVOD	NA	Not validated
Naal et al^58^	Hypochloraemia (≤100 mmol/liter)	Serum	Prognosis	IPAH, HPAH	0.69	Not validated
Prins et al^59^	Hypochloraemia (≤101 mmol/liter)	Serum	Diagnosis, monitoring	PAH (diverse groups)	0.64	Not validated
Xanthouli et al^60^	Hypochromic erythrocytes (>2%)	Blood	Prognosis	IPAH, APAH	NA	Not validated
Bouzina et al^61^	IGFBP-1	Plasma	Diagnosis, prognosis, monitoring	PAH (majority IPAH, APAH-SSc)	NA	Not validated
Yang et al^62^	IGFBP-2	Serum	Diagnosis, prognosis, monitoring	Majority IPAH, APAH-CTD	0.76	Externally validated
Di Benedetto et al^63^	IL-32, IL-32+ cells	Serum	Diagnosis	APAH-SSc	0.95	Not validated
Hirsch et al^64^	IL-6, IP-10, NT-proBNP, PlGF, TNF-b	Serum	Prognosis	IPAH, APAH-CTD, APAH-CHD, drug-induced PAH	NA	Externally validated
Matura et al^65^	IL-6, TNF-α	Plasma	Monitoring	PAH (majority IPAH and APAH-CTD)	NA	Not validated
Kopeć et al^66^	LDL-C	Plasma	Prognosis	IPAH, APAH-CHD, APAH-CTD	NA	Not validated
Omura et al^67^	Long noncoding RNA H19 (H19)	Plasma	Diagnosis, prognosis, monitoring	IPAH	0.55-0.83	Externally validated
Chen et al^68^	26 and 15 metabolites, including lysophosphatidylcholine, and perillic acid	Plasma	Diagnosis	IPAH, APAH-CHD	0.74-0.99	Not validated
Kheyfets et al^69^	Machine learning algorithm, including (HGF, IL-2, IL-9, lymphocytes (%, *n*), NT-proBNP, SCF)	Plasma	Prognosis	IPAH/HPAH, APAH	0.64-0.72; 0.81, 0.94^g^	Internally and externally validated
Rhodes et al^70^	MiR-150	Plasma, microvesicles, blood cells	Prognosis	IPAH (majority)	0.75-0.85	Externally validated
Huang et al^71^	MiR-596	Plasma	Prognosis	IPAH	0.86	Internally validated
Errington et al^72^	Up to 20 MiR:s, including MiR-636, MiR-187-5p, NT-proBNP	Plasma	Diagnosis	IPAH, APAH-SSc	0.78-0.91, 0.79-0.97^h^	Externally validated
Wetzl et al^73^	MMP-2/TIMP4 ratio	Plasma	Prognosis, monitoring	IPAH	0.82, 0.92^i^	Not validated
Arvidsson et al^74^	MMP-7	Plasma	Diagnosis	PAH (majority IPAH, APAH-SSc)	0.75	Not validated
Zhang et al^75^	NOx	Plasma	Monitoring, prognosis	IPAH	0.76	Not validated
Rhodes et al^76^	Neuropilin 1, NT-proBNP, peroxiredoxin-4, peroxidasin, renin, SVEP1, thrombospondin-2	Plasma	Prognosis, monitoring	IPAH/HPAH, drug-induced PAH	0.73-0.84	Externally validated
Chin et al^77^	NT-proBNP	Plasma	Prognosis, monitoring	IPAH, HPAH, APAH-CTD, APAH-CHD, drug-induced PAH	NA	Not validated
Simpson et al^78^	NT-proBNP, ST2	Serum	Prognosis, monitoring	PAH (majority IPAH and APAH-CTD)	0.72-0.73	Not validated
Tiede et al^79^	PlGF, sFlt-1	Plasma	Diagnosis	IPAH, APAH-CTD, APAH-CHD	0.66-0.91	Not validated
Kylhammar et al^80^	PlGF, sFlt-1, TNF-α, VEGF-D	Plasma	Susceptibility/risk prediction, monitoring	APAH-SSc, IPAH	NA	Not validated
Arvidsson et al^81^	Prolargin	Plasma	Diagnosis	PAH (majority IPAH, APAH-SSc)	0.84	Not validated
Säleby et al^82^	RET	Plasma	Diagnosis	PAH (majority IPAH, APAH-SSc)	NA	Not validated
Bouzina et al^83^	SCF, TGF-α	Plasma	Monitoring	PAH (majority IPAH, APAH-SSc)	0.75	Not validated
Rhodes et al^84^	Score derived from 25 RNAs	Whole blood	Diagnosis, prognosis, monitoring	IPAH, HPAH	0.87	Externally validated
Kikuchi et al^85^	Selenoprotein P	Serum	Diagnosis, prognosis, monitoring	IPAH, HPAH, APAH-CTD, APAH-CHD	0.83	Not validated
Zheng et al^86^	ST2	Plasma	Prognosis, monitoring	IPAH	0.79	Not validated
Wang et al^87^	Uric acid, ∆uric acid	Serum	Monitoring, prognosis	APAH-CTD	NA	Not validated
Chen et al^88^	YKL-40	Plasma	Prognosis	IPAH	0.68, 0.72^j^	Not validated

Abbreviations: AUC, area under the receiver operating characteristic curve; NA, not available. Pulmonary hypertension–specific abbreviations: APAH-CHD, congenital heart disease–associated pulmonary arterial hypertension; APAH-CTD, connective tissue disease–associated pulmonary arterial hypertension; APAH-SSc, systemic sclerosis–associated pulmonary arterial hypertension; APAH-SLE, systemic lupus erythematosus–associated pulmonary arterial hypertension; HPAH, heritable pulmonary arterial hypertension; IPAH, idiopathic pulmonary arterial hypertension; PoPH, porto-pulmonary hypertension; PVOD, pulmonary veno-occlusive disease. Biomarker-specific abbreviations: ACE2, angiotensin-converting enzyme 2; ADAMTS13, a disintegrin and metalloproteinase with thrombospondin type 1 motif, member 13; ADMA, asymmetric dimethylarginine; ALT, alanine aminotransferase; AMBP, alpha-1-microglobulin/bikunin precursor; Anti-AT1R-antibodies, antiangiotensin type 1 receptor antibodies; anti-ETAR antibodies, antiendothelin-1 type A receptor antibodies; AST, aspartate transferase; BMP-9, bone morphogenetic protein-9; BNP, brain natriuretic peptide; CCL21, C-C motif chemokine ligand 21; CEACAM8, carcinoembryonic antigen-related cell adhesion molecule 8; CFD, complement factor D; CFH, complement factor H; CTGF, connective tissue growth factor; CXCL1, CXC motif chemokine ligand 1; CXCL10, CXC motif chemokine ligand 10; CXCL12, CXC motif chemokine ligand 12; CXCL4, CXC motif chemokine ligand 4; CXCL8, CXC motif chemokine ligand 8; CXCL9, CXC motif chemokine ligand 9; FAHFA, fatty acyl esters of hydroxy fatty acid; FGF-23, fibroblast growth factor-23; FSTL3, follistatin-Like 3; GDF-15, growth differentiation factor 15; GGT, γ-glutamyltransferase; HDGF, hepatoma-derived growth factor; HDL, high-density lipoprotein; HDL-4-Apo A-1, HDL apolipoprotein A-1; HDL-4-Apo A-2, HDL apolipoprotein A-2; HGF, hepatocyte growth factor; IGFBP-1, insulin-like growth factor-binding protein 1; IGFBP-2, insulin-like growth factor-binding protein 2; IGFBP-7, insulin-like growth factor-binding protein 7; IL-2, interleukin-2; IL-6, interleukin-6; IL-9, interleukin-9; IL-32, interleukin-32; IP-10, interferon gamma-induced protein 10; LDL-C, low-density lipoprotein cholesterol; LOX1, lectin-like oxidized low-density lipoprotein receptor-1; LPL, lipoprotein lipase; LTB4, leukotriene B4; MiR-150, micro-RNA 150; MiR-596, micro-RNA 596; MiR-636, Micro-RNA 636; MiR-187-5p, Micro-RNA 187-5p; MMP-2, matrix metalloproteinase 2; MMP-7, matrix metalloproteinase-7; MR-proADM, mid-regional proadrenomedullin; NLR, neutrophil-to-lymphocyte ratio; NOx, nitrogen oxides; NT-proBNP, N-terminal prohormone of brain natriuretic peptide; PlGF, placental growth factor; RAGE, receptor for advanced glycation endproducts; RET, receptor tyrosine kinase RET; RNASE1, ribonuclease A family member 1; SCF, stem cell factor; sFlt-1, soluble fms-like tyrosine kinase-1 or soluble VEGF receptor-1 (sVEGFR-1); ß-NGF, beta-nerve growth factor; ST2, soluble suppression of tumorigenicity 2 or interleukin 1 receptor, type I (IL1R1); SVEP1, Sushi, von Willebrand factor type A, epidermal growth factor and pentraxin domain containing 1; TGF-α, transforming growth factor alpha; TIMP1, tissue inhibitor of metalloproteinase 1; TIMP2, tissue inhibitor of metalloproteinase 2; TIMP4, tissue inhibitor of metalloproteinase 2; TNF-α, tumor necrosis factor alpha; TNF-β, tumor necrosis factor beta; TRAIL, tumor necrosis factor-related apoptosis-inducing ligand; VEGF-D, vascular endothelial growth factor D; vWF, von Willebrand factor; YKL-40, chitinase-3-like protein 1 or cartilage glycoprotein 39.

Population implies the subgroup/s in which the biomarker candidates can be used for. Validation implies validation within the same study, unless stated otherwise. The reported AUCs may not represent all clinical classes provided for each of biomarker candidate(s). The reported AUCs imply the reported ranges of the uni- and/or multivariable/adjusted AUCs.

a AUC for annexin A-1.

b AUC (0.74) for the panel score change from baseline to follow-up, after treatment initiation.

c Estimated visually from curves as the exact AUCs were not stated.

d 0.99 in distinguished PoPH from control subjects, PAH, and group 2 and 3 PH.

e 0.68 in identifying PAH, and 0.88 in predicting mortality.

f 0.70 for NT-proBNP and 0.77 for GGT.

g 0.64 to 0.72 of biomarker candidates univariably, and 0.81 and 0.94 multivariably in the internal- and external validation cohorts, respectively.

h 0.78 to 0.91 when combining up to 20 MiR:s in 4 separate machine learning methods, or by using MiR-636 or MiR-187-5p separately. 0.79-0.97 when adding NT-proBNP to the machine learning models in identifying PAH from healthy and disease controls (no PH).

i 0.82 in predicting clinical worsening and 0.92 in predicting mortality.

j 0.68 in predicting clinical worsening and 0.72 in predicting mortality.

## Biomarkers in PAH and definitions

The clinical demand for thoroughly characterized biomarkers in PAH is increasing to facilitate the incorporation of precision medicine and improve outcomes.^16^, ^18^, ^89^ Beyond the use of natriuretic peptides,^5^ the creatinine estimate of glomerular filtration rate in the REVEAL-based risk scores,^89^, ^90^ as well as uric acid and the anticentromere antibody profile as a part of the multimeric DETECTion of PAH in systemic sclerosis (SSc) (DETECT) algorithm,^91^ other emerging blood-borne biomarkers in PAH are yet to be translated clinically.^4^ Through a joint effort, the U.S. Food and Drug Administration (FDA) and the National Institutes of Health biomarker working group established the Biomarkers, EndpointS, and other Tools resource to promote clarity in communication and harmonize nomenclature used in the biomarker field across different communities; with the ultimate goal to enable efficient clinical translation of scientific discoveries.^17^ A biomarker is “a defined characteristic that is measured as an indicator of normal biological processes, pathogenic processes, or biological responses to an exposure or intervention, including therapeutic interventions,” but is not a clinical outcome assessment, that is, how an individual feels, functions, or survives.^17^

In PAH, several classes of blood-borne biomarker candidates acknowledged in numerous mechanistic pathways^4^, ^5^ are emerging, belonging to different biological entities, including genetics,^4^ transcripts,^38^, ^70^, ^71^, ^72^, ^84^ metabolites,^19^, ^20^, ^56^, ^68^, ^75^ and proteins.^18^, ^44^, ^76^ Clinically and in concordance with the Biomarkers, EndpointS, and other Tools glossary, and European Medicines Agency definitions,^17^, ^92^ these classes comprise mainly biomarkers used in PAH: (1) diagnosis and differentiation, (2) prognosis, (3) monitoring of (risk strata, structural changes, hemodynamic- and treatment responses), and (4) susceptibility/risk prediction, summarized in (Table 2).

### Diagnostic and differentiative biomarkers in PAH

Several types of diagnostic biomarkers have been explored, including discrimination of PAH from healthy controls,^19^, ^39^, ^54^, ^72^, ^84^ differentiation of PAH from other subgroups of PH and dyspnea,^22^, ^24^, ^26^, ^27^, ^28^, ^48^, ^61^, ^74^, ^81^, ^82^, ^83^ as well as in identifying a specific subgroup from other PAH subgroups (Table 2).^19^, ^32^, ^53^

In a recent study including >1,400 patients, Alotaibi et al investigated >689 circulating bioactive lipid metabolites in differentiating idiopathic PAH (IPAH) from SSc-associated PAH (APAH-SSc), comprising independent discovery- and validation cohorts.^19^ Nine lipid metabolites were found to distinguish IPAH from APAH-SSc with an AUC (as a measure of accuracy) of 0.84 to 0.86, independent of age, sex, body mass index, and 6-minute walk distance (6MWD).^19^ Furthermore, by using independent cohorts of APAH-SSc and SSc without PH, 5 of the 9 metabolites were found increased in PAH independent of the presence of SSc and were associated with parameters of disease severity.^19^

Bauer et al further identified a panel of 8 biomarkers superior to N-terminal prohormone of brain natriuretic peptide (NT-proBNP) alone, which could identify patients with APAH-SSc from SSc without PH with an AUC of 0.74. More studies are, however, needed to evaluate the panel in a prospective, longitudinal setting and its incremental value to the current DETECT algorithm.^39^

In another study, Yang et al found that elevated serum hepatoma-derived growth factor (HDGF) levels identified PAH from healthy (AUC of 0.89) and chronic, multimorbid disease controls.^54^ HDGF also correlated with 6MWD and functional class and emerged as a strong predictor of mortality independent of age, PAH etiology, invasive hemodynamics, and NT-proBNP.^54^ In the follow-up multicenter study, HDGF was confirmed to be a predictor of mortality and disease severity.^53^ However, once adjusted for age and sex, elevated HDGF levels were associated only with IPAH and porto-pulmonary hypertension (PoPH), with no significant associations found across the other PAH subtypes.^53^ A limitation of the follow-up study cohort was that the blood samples were not collected contemporarily with other clinical parameters as in the first study, which may have impacted the results.^53^ Nonetheless, further validation of the clinical applicability of HDGF in an appropriate cohort is warranted.^54^, ^53^ In addition to HDGF, in a study by Nikolic et al, plasma bone morphogenetic protein-9 emerged as a differentiative biomarker of PoPH among patients with liver disease, other PAH and PH etiologies, as well as a predictor of transplant-free survival in PAH.^32^

Intriguingly, by conducting a whole-blood RNA sequencing in idiopathic- and heritable PAH, Rhodes et al identified 507 transcripts that were differentially expressed in patients with PAH compared to healthy controls.^84^ Furthermore, a score derived from a signature profile of 25 RNAs, identified PAH from healthy controls with an AUC of 0.87, correlated with clinical risk and disease severity measures (6MWD, brain natriuretic peptide (BNP), or NT-proBNP, and functional class) and long-term survival.^84^

In a series of studies targeting diverse mechanistic pathways recognized in the pathobiology of PH, several biomarker classes were assessed, including the diagnostic potential of various plasma proteins in identifying PAH from controls (healthy and diseased) and other PH subgroups.^22^, ^24^, ^26^, ^28^, ^48^, ^61^, ^74^, ^81^, ^82^, ^83^ The diagnostic/differentiative candidates included prolargin,^81^ proto-oncogene tyrosine-protein kinase receptor Ret (RET),^82^ matrix metalloproteinase-7,^74^ insulin-like growth factor-binding protein 1 (IGFBP-1),^61^ and tumor necrosis factor-related apoptosis-inducing ligand (TRAIL).^28^ Specifically, in 1 study investigating inflammatory and coagulation markers, Ahmed et al found that elevated ADAMTS13 differentiated treatment naïve patients with PAH from other PH subgroups and patients with dyspnea without PH with an AUC of 0.91, adjusted for age and sex.^24^

Another aspect of particular importance is the unabated increase of elderly, multimorbid PAH population in the international western registries,^5^, ^93^, ^94^ complicating the hemodynamic and clinical distinction between PAH and those with PH associated with heart failure with preserved ejection fraction (HFpEF).^26^, ^95^ In one of the studies investigating cancer and metabolism-related proteins, Ahmed et al found that 3 biomarkers (alpha-1-microglobulin/bikunin precursor, lipoprotein lipase, and glyoxalase I (adjusted for age and sex)) alone, or in combination differentiated HFpEF-PH from PAH with an AUC of 0.87.^26^

### Prognostic biomarkers in PAH

**Figure 2:**
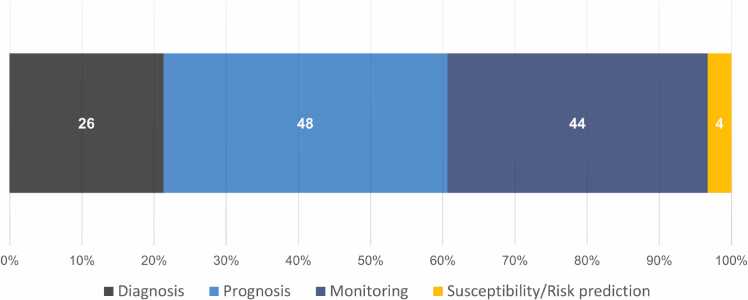
Distribution of the number of studies investigating different circulating biomarker classes in pulmonary arterial hypertension (2012-2023). In the present synthesis, each study (Table 2) may have been included more than once.

Over the past decade, prognosis has been the primary focus of circulating biomarker studies in PAH (Figure 2).^18^, ^21^, ^22^, ^23^, ^24^, ^27^, ^28^, ^29^, ^30^, ^31^, ^32^, ^33^, ^34^, ^35^, ^36^, ^38^, ^40^, ^41^, ^45^, ^46^, ^47^, ^51^, ^52^, ^53^, ^54^, ^55^, ^56^, ^57^, ^58^, ^60^, ^61^, ^62^, ^66^, ^67^, ^69^, ^70^, ^71^, ^73^, ^75^, ^76^, ^77^, ^78^, ^84^, ^85^, ^86^, ^87^, ^88^, ^96^, ^97^ Typically, prognostic biomarkers inform the odds/risk for developing a broad range of complications, including mortality and survival, transplantation, decompensation, and/or hospitalizations.^17^, ^18^, ^21^, ^22^, ^23^, ^24^, ^27^, ^28^, ^29^, ^30^, ^31^, ^32^, ^33^, ^34^, ^35^, ^36^, ^38^, ^40^, ^41^, ^45^, ^46^, ^47^, ^51^, ^52^, ^53^, ^54^, ^55^, ^56^, ^57^, ^58^, ^60^, ^61^, ^62^, ^66^, ^67^, ^69^, ^70^, ^71^, ^73^, ^75^, ^76^, ^77^, ^78^, ^84^, ^85^, ^86^, ^87^, ^88^, ^96^, ^97^ Notably, the distinction between prognostic and predictive biomarkers can sometimes be difficult and challenging.^17^ In fact, some biomarkers may both be predictive and prognostic.^17^ A predictive biomarker informs the overall expected effect of a therapeutic intervention solely in biomarker-defined patients and may itself be a target for therapy, for example, genetic mutations in various malignancies.^17^, ^98^, ^99^

BNP and NT-proBNP, alone or in combination with other surrogates of outcome measures/risk assessment tools, remain the most validated and widely used prognostic biomarkers in routine clinical practice of PAH.^5^, ^33^, ^41^, ^51^, ^55^, ^69^, ^76^, ^77^, ^78^, ^96^ In a posthoc analysis of the Prostacyclin [PGI2] Receptor Agonist In Pulmonary Arterial Hypertension (GRIPHON) trial, Chen et al confirmed the highly prognostic potential of NT-proBNP cut-off levels (<300 ng/liter, 300-1,400 ng/liter, and >1,400 ng/liter) used in the 3 strata ESC/ERS risk assessment strategy, at baseline and follow-up.^77^ Moreover, Frantz et al, in a large study based on data from REVEAL, demonstrated that in PAH, baseline BNP levels (≤340 pg/ml and >340 pg/ml) strongly predicted survival up to 5 years, and BNP level changes from latest assessment within 1- and 5-years postenrollment to baseline correlated with the mortality risk.^33^

Apart from natriuretic peptides which are indicators of ventricular stress, there is a paucity of markers addressing the adverse right ventricular remodeling in PAH.^67^ Interestingly, in a recent study by Omura et al, long noncoding RNA H19 appeared to be a marker of maladaptive right ventricular coupling, differentiating controls with normal right ventricular function from IPAH, as well as IPAH with the decompensated right ventricle phenotype vs the compensated ditto.^67^ H19 also emerged as a promising predictor of event-free survival in patients with IPAH and was proposed as a potential therapeutic target of maladaptive right ventricular remodeling, as supported by the additional in vivo knock-down findings.^67^

In a retrospective posthoc analysis of the Randomized Clinical Trial of Aspirin and Simvastatin for Pulmonary Arterial Hypertension (ASA-STAT), Al-Naamani et al found that at baseline, lower total cholesterol and von Willebrand factor (vWF) levels, as well as higher plasma NT-proBNP, were predictors of death or lung transplantation, after adjustment for age, sex, PAH etiology, and 6MWD.^55^ Subsequently, in one of the studies by Ahmed et al, plasma vWF was confirmed to be a marker of prognosis, discriminating survivors vs nonsurvivals. Moreover, adjusted for age, sex, and combined with the ESC/ERS risk score, vWF predicted higher mortality rates with an AUC of 0.94.^24^ Other prognostic biomarker candidates in PAH identified/confirmed by Arvidsson et al, Ahmed et al and/or Bouzina et al include plasma annexin A-1,^28^ IGFBP-1,^61^ and adrenomedullin.^22^

Through a metabolomics approach, Harbaum et al investigated lipoprotein profiles in patients with idiopathic and heritable PAH, and demonstrated that high-density lipoprotein apolipoprotein A-1(HDL-4-Apo A-1), HDL-4-Apo A-2, and HDL-4-phospholipids were predictors of survival, independent of established outcome measures, including NT-proBNP and 6MWD.^56^ Intriguingly, the same group conducted an integrated analysis of the plasma genome and proteome in another large, multicenter study of patients with heritable and idiopathic PAH.^47^ Apart from differentiating PAH from healthy controls, 8 plasma proteins emerged as predictors of long-term survival and exhibited genetic control in PAH, of which netrin-4 and thrombospondin-2 were proposed to be directly implicated in the pathobiology of PAH.^47^

In a multicenter study comprising 4 independent cohorts of idiopathic and heritable PAH, Rhodes et al, through a high-throughput proteomic analysis, demonstrated that a score derived from a panel of 9 plasma proteins, identified patients at high mortality risk.^18^ Additionally, the protein-derived score improved the prognostic accuracy of NT-proBNP and the REVEAL equation.^18^

In a study by Simpson et al, serum endostatin—an angiostatin circulating peptide, previously assessed in vivo by the same group,^100^ was investigated anew, in a large, multicenter study of patients with PAH, to evaluate its prognostic and incremental value to contemporary risk assessment tools.^45^ The authors showed that the age- and sex-adjusted serum endostatin levels were associated with hemodynamics and clinical variables, and that higher levels were independently associated with survival, improving the predictive accuracy of ESC/ERS- and REVEAL-based risk assessment strategies.^45^ Using the same cohort of patients with PAH, Simpson et al further investigated the prognostic potential of soluble suppression of tumorigenicity 2 (ST2) and galectin 3—recently FDA-approved biomarkers in noninvasive risk stratification of left heart failure—along with NT-proBNP.^78^ Apart from confirming the prognostic value of NT-proBNP in PAH, increased serum ST2 proved to be a strong predictor of mortality independent of NT-proBNP, and improved the predictive capacity of REVEAL 2.0 and the separation of different risk groups.^78^ In contrast, galectin 3 correlated weakly to modestly with cardiac hemodynamics, and after adjustment for clinical variables, showed no consistent correlation with survival.^78^ Although multicenter and large, further validation, especially of galectin 3 is required, as the blood sample collection was not contemporaneous with the assessments of other clinical variables.^78^, ^45^

Already established markers of other conditions have also been investigated in relation to prognosis in PAH. For instance, Yogeswaran et al studied readily and routinely available laboratory parameters in PAH and chronic thromboembolic PH, and found that a score derived from γ-glutamyltransferase, aspartate aminotransferase/alanine aminotransferase ratio, and the neutrophil-to-lymphocyte ratio predicted mortality risk, in PAH or chronic thromboembolic PH, with an accuracy comparable to ESC/ERS-derived risk scores.^29^ In another study, Xanthouli et al found that the presence of >2% hypochromic erythrocytes in PAH patients predicted the time to clinical worsening and survival, independent of hemoglobin deficiency, low iron-, and low ferritin levels.^60^

Finally, in light of the emergence of new PAH therapies, particularly in relation to Sotatercept—a fusion protein that traps activins and growth factors leading to reversal of vascular remodeling^101^—Guignabert et al found that elevated serum activin A and follistatin-like 3, key members of the activin pathway, predicted death or lung transplantation in patients with idiopathic, heritable, or anorexigenic-associated PAH, independent of other noninvasive parameters currently used for PAH risk assessment (New York Heart Association Functional Classification, 6MWD, and NT-proBNP).^23^

### Monitoring biomarkers in PAH

This entity refers to biomarkers that at time of assessment (1) directly reflect and correlate with outcome measures including disease severity, hemodynamic deterioration, or inadequate treatment response and/or (2) have the potential to directly impact clinical management.^17^ As the current 2022 ESC/ERS PH guidelines recommend prognostic risk stratification to guide treatment in PAH,^5^ biomarkers reflecting risk strata were defined in the present review as both monitoring and prognostic.

A number of the aforementioned biomarker candidates have also been investigated in monitoring of PAH, including in risk assessment.^18^, ^45^, ^53^, ^54^, ^55^, ^77^, ^78^ For instance, changes in the prognostic score derived from 9 plasma proteins after treatment initiation in idiopathic and heritable PAH, identified patients who did not adequately respond to treatment.^18^ Furthermore, in the ASA-STAT trial, vWF, HDL, and thromboxane B_2_ were associated with worse World Health Organization (WHO)-functional class after adjustment for age, sex, and PAH etiology.^55^

The current 2022 ESC/ERS PH guidelines endorse multiparametric risk stratification to guide treatment in PAH (recommendation class I, level of evidence B), with the goal to achieve or maintain a low-risk profile corresponding to a 1-year mortality <5%.^5^, ^102^ Most validated risk assessment instruments have AUCs (as a measure of prognostic accuracy) ranging between 0.62 and 0.76, implying room for further improvement.^89^, ^103^, ^104^ In a highly characterized PAH population, Kikuchi et al showed that serum selenoprotein P changes correlated with invasive hemodynamic improvements in response to treatment.^85^ Moreover, its levels discriminated PAH vs non-PH controls, and predicted outcomes at baseline and follow-up.^85^ In the posthoc analysis of the GRIPHON trial, Chen et al found that NT-proBNP levels were associated with treatment response to selexipag.^77^

Boucly et al conducted a systemic analysis of a well-phenotyped, treatment naïve, and longitudinally followed PAH cohort, and found that a biomarker panel comprising β-nerve growth factor, CXC motif chemokine ligand 9 (CXCL9), and TRAIL, was independently associated with prognosis at baseline and first follow-up after treatment initiation.^30^ In a multivariable analysis, the 3 biomarkers proved to be superior to 6MWD, BNP/NT-proBNP, and WHO-functional class in predicting prognosis, and were proposed to be considered in concurrent risk stratification strategies to guide treatment.^30^ In a large, multicenter study, Rhodes et al assessed the plasma proteome and identified 31 proteins that predicted survival independent of age, sex, NT-proBNP, and 6MWD.^76^ Thereafter, a score model derived from 6 of the 31 proteins was constructed that complemented NT-proBNP and established clinical risk factors in predicting the 5-year mortality risk. This model improved the AUC from 0.76 to 0.82 when combined with NT-proBNP compared to NT-proBNP alone, and may thus have implications for evaluating treatment response.^76^

Although incompletely established mechanism by which the extracellular primary release occur, circulating cell-free DNA (cfDNA) is emerging as a biomarker of cell injury and cell turnover, rejection of solid organ transplants, and in surveillance in oncology, reflecting worse prognosis in diverse conditions.^105^, ^106^, ^107^, ^108^ Intriguingly, Brusca et al in a study of PAH patients found that cfDNA levels predicted death or transplant-free survival independent of age, sex, and REVEAL 2.0 risk score, and correlated with disease severity, improving risk discrimination in REVEAL 2.0 (AUC from 0.72-0.78). Although further validation is warranted, serial analysis of cfDNA may be a promising marker to surveil disease severity in PAH.^36^

In recent years, patient-reported outcomes have gained more importance clinically, yielding information that cannot be obtained through objective clinical assessments alone, and with the potential to provide independent prognostic information.^5^, ^65^, ^109^ Hence, biomarkers reflecting patient-reported outcomes with pathological linkage are of particular interest.^65^ In a follow-up study of the ASA-STAT trial, Matura et al found that the inflammatory markers interleukin-6 and tumor necrosis factor-α (TNF-α) were associated with the Medical Outcomes Study Short Form-36 score and its subscales comprising pain, vitality, and mental health (anxiety/depression) at baseline and the 3-month follow-up.^65^ Elevated and adjusted interleukin-6 levels were associated lower Study Short Form-36 scores indicating worse pain, vitality, and mental health, whereas elevated and adjusted TNF-α levels were associated with improved quality of life–related symptoms of vitality and mental health, but surprisingly also with increased pain.^65^

### Susceptibility/risk biomarkers in PAH

**Figure 3:**
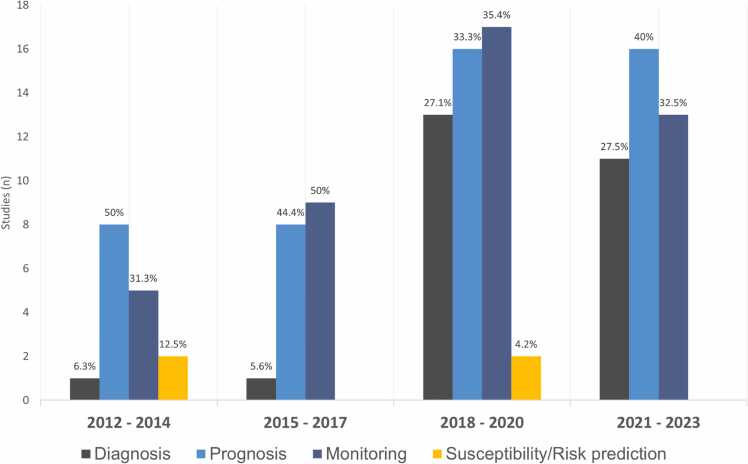
Overview of the distributions and trends of the number studies exploring different circulating biomarker classes in pulmonary arterial hypertension, stratified by 3-year intervals (2012-2023). In the present synthesis, each study (Table 2) may have been included more than once.

Susceptibility biomarkers identify individuals at higher risk of developing a disease compared to those without the biomarker.^17^ For instance, numerous genetic mutations associated with increased susceptibility to develop specific PAH phenotypes have been identified.^5^ Nonetheless, beyond genetic mutations, there is an overall paucity of studies exploring susceptibility biomarkers in PAH (Table 2). Additionally, most available studies have investigated biomarkers in predicting the occurrence of PAH or fibrosis in SSc (Figure 3).^35^, ^39^, ^44^, ^80^ In a large multicenter study, Becker et al demonstrated that serum antiendothelin receptor type A and antiangiotensin type 1 receptor antibodies not only differentiated APAH-SSc from PAH subgroups and SSc without PH, but also predicted mortality in APAH-SSc, as well as the development of PAH in an independent, longitudinally followed cohort of SSc.^27^ Although awaiting replication in another study, van Bon et al conducted a proteome-wide analysis and found that high circulating levels of CXCL4 correlated with the occurrence of PAH and predicted the development of fibrosis and PAH in patients with SSc.^44^ Likewise, in a large study by Hoffmann-Vold et al, serum C-C motif chemokine ligand 21 (CCL21) emerged as an independent predictor of PAH development in SSc.^35^

## Gaps in evidence and challenges

Although advances have been made in identifying novel biomarkers, their increasing numbers may appear deluging (Table 2). It is therefore crucial to thoroughly evaluate new biomarker candidates as they emerge.^17^, ^99^ First, there is a paucity of studies in PAH addressing granular distinctions of biomarker levels in relation to outcome and PAH-subtype,^96^ which may partially be explained by that repositories enabling such studies may be difficult, and slow to establish, especially if originating from small PH centers. Moreover, the analyses of PAH subgroups in aggregates may not only obscure the discovery of significant subgroup-specific associations but may also drive statistical significance in the aggregate group, yielding results that may not be generalizable for all included subgroups.^96^ Second, the steadily increasing number of elderly and multimorbid patients with PAH encourages the inclusion of not only negative (healthy) control groups, but also positive (diseased) control cohorts (patients with cardiopulmonary disease/comorbidity without recognized PH presenting with unspecific symptoms or signs including dyspnea at rest or exertion, fatigue, chest pain/discomfort, edema, presyncope/syncope—initial symptoms that may appear in patients with PAH) relevant to the purpose of the candidate biomarker(s).^5^, ^93^, ^94^, ^110^ A subgroup of the diseased controls may develop PH or PAH in the future, for example, patients with SSc without PAH or patients with left heart disease without contemporaneous PH. Ideally, hemodynamic assessment should be available for the positive control cohorts when included. Third, biomarkers facilitating clinical decision-making,^111^, ^112^ adding to already existing algorithms,^26^ such as in identifying high-risk individuals requiring fast-track PH evaluation,^5^ reflecting quality of life outcomes^65^ as well as in distinction of PH subgroups and phenotypes, for example, HFpEF associated with PH from PAH,^26^, ^95^ are warranted. Fourth, the journey from discovering a novel biomarker candidate to its validation and clinical implementation can be lengthy and requires an understanding of its pharmacokinetics, pharmacodynamics, and diurnal variations. The candidate biomarker also necessitates independent analytical and clinical validation, preferably in prospective and longitudinal cohorts, to determine whether the biomarker and the analytical method are fit-for-purpose.^17^, ^67^ Furthermore, evaluation of existing and clinically approved biomarkers of other diseases or conditions in PAH is also needed and may be of great value for the rapid clinical integration of clinically appropriate candidates, allowing for improved health care, especially in low-income countries.^29^, ^57^, ^58^, ^59^ Finally, negative results should be made publicly available, as scientific advance across all disciplines relies on corroboration, reproducibility, and availability of research results.^113^ Additionally, cost-effectiveness analyses are encouraged of candidate biomarkers.

## Future directions and concluding remarks

In recent years, significant advances have occurred in the development high-throughput analytical methods and informatics.^114^ This has enabled a feasible way of measuring millions of data points simultaneously and the generation of large biological data sets (omics), driving a paradigm shift toward the way of approaching biomarkers.^114^ In line with these advances, computational methods using artificial intelligence and machine learning are emerging, providing an unparalleled asset in therapeutic target- and biomarker discovery.^72^, ^111^, ^114^ Notwithstanding, in PAH, several attempts have already been made toward the integration of different omics modalities (e.g., genomics, transcriptomics, proteomics, and metabolomics), into already existing algorithms and risk assessment tools, yielding novel molecular- and biomarker-specific outcome associations.^47^, ^56^, ^84^, ^115^, ^116^ However, unless replicated and appropriately followed by, for example, in vivo and in vitro studies, omics studies alone cannot elucidate underlying mechanisms of action.^116^

Considering the abovementioned, we believe that a number of biomarkers should be considered for incorporation into the routine clinical practice in near future, especially those with existent regulatory approvals, for example, assay approvals, and those already used in other diseases/conditions. Consequently, several biomarkers, presented in Table 3, have been selected as promising candidates for potential clinical use, further validation, and incorporation into the future. The selection was based on (1) feasibility for clinical implementation, as incorporation is facilitated for biomarkers already in clinical use with approved analytical methodologies; (2) internally and/or externally validated biomarkers confirming their clinical associations; (3) the relative clinical importance of the implementation of the biomarker, that is, the added value and the estimated impact on clinical practice, along with AUCs, and the size of study population. Future guidelines should promote clinically promising candidate biomarkers to further direct the analytical development and optimization, as well as facilitate the currently stagnant prospective validation of the candidate biomarkers for clinical integration including ST2, activin A, follistatin-like 3, vWF, total cholesterol, interleukin-6, and TNF-α, consistent with the FDA and the European Medicines Agency regulations.^17^, ^92^, ^117^

**Table 3:** Promising Candidate Biomarkers That Should Be Considered for Further Validation and Possible Incorporation in the Routine Practice in PAH

Biomarker candidate (s)/panel	Biospecimen	Clinical use
Collagen IV, endostatin, IGFBP-2, IGFBP-7, MMP-2, neuropilin 1, NT-proBNP, RAGE^a^	Serum	Identifying PAH in a mixed population of patients with systemic sclerosis.^39^
ADAMTS13^a^	Plasma	Identifying PAH from other dyspnea groups and healthy controls.^24^
RET	Plasma	Identifying PAH from other dyspnea groups and healthy controls.^82^
TRAIL	Plasma, serum	Identifying PAH from other dyspnea groups and healthy controls.^28^ Survival and treatment response in PAH.^30^
AMBP, glyoxalase I, LPL	Plasma	Differentiating HFpEF with pulmonary hypertension from PAH.^26^
ST2^a^	Plasma, serum	Predictor of mortality and clinical worsening in PAH.^78^, ^86^
Endostatin	Plasma	Predictor of survival and hemodynamic monitoring in PAH.^45^, ^46^
Apolipoprotein E, CFD, CFH, erythropoietin, IGFBP-1, ST2, plasminogen, TIMP1, TIMP2^a^	Plasma	Predictors of mortality and treatment response/risk stratification of idiopathic- and heritable PAH.^18^
Long noncoding RNA H19	Plasma	Identifying maladaptive right ventricular coupling/function in IPAH, and vs controls. Predictor of event-free survival in IPAH.^67^
von Willebrand factor^a^	Plasma	Predictor of mortality^28^, ^55^ and lung transplantation in PAH.^55^
Total cholesterol, thromboxane B_2_^a^	Plasma	Predictors of mortality and lung transplantation in PAH.^55^
HDL^a^	Plasma	Associated with WHO-functional class in PAH.^55^
HDL-4-Apo A-1, HDL-4-Apo A-2, HDL-4-phospholipids	Plasma	Predictor of survival in idiopathic- and heritable PAH.^56^
Activin A and follistatin-like 3	Plasma	Predictors of death or lung transplantation in idiopathic-, heritable-, and anorexigenic-related PAH.^23^
β-nerve growth factor, CXC motif chemokine ligand 9	Serum	Predictors of death or transplantation at baseline and after treatment initiation.^30^
Cell-free DNA	Plasma	Predictor of mortality and transplant-free survival, surveillance of disease severity in PAH.^36^
Interleukin-6 and TNF-α^a^	Plasma	Associated with quality of life and mental health in PAH.^65^
GGT, AST/ALT ratio, and NLR^a^	Blood	Predictor of mortality risk/risk stratification in PAH.^29^
Hypochromic erythrocytes (>2%)^a^	Blood	Predictor of time to clinical worsening and survival in PAH.^60^
Antiendothelin receptor type A and antiangiotensin type 1 receptor antibodies	Serum	Predictor of mortality and PAH development in systemic sclerosis.^27^
CXC motif chemokine ligand 4	Plasma	Predictor of fibrosis and PAH development in systemic sclerosis.^44^
C-C motif chemokine ligand 21	Serum	Predictor of PAH development in systemic sclerosis.^35^

Abbreviations: HFpEF, heart failure with preserved ejection fraction; PAH, pulmonary arterial hypertension; WHO, World Health Organization. Biomarker-specific abbreviations: ADAMTS13, a disintegrin and metalloproteinase with thrombospondin type 1 motif, member 13; ALT, alanine aminotransferase; AMBP, alpha-1-microglobulin/bikunin precursor; AST, aspartate transferase; CCL21, C-C motif chemokine ligand 21; CFD, complement factor D; CFH, complement factor H; GGT, γ-glutamyltransferase; HDL, high-density lipoprotein; IGFBP-1, insulin-like growth factor-binding protein 1; IGFBP-2, insulin-like growth factor-binding protein 2; IGFBP-7, insulin-like growth factor-binding protein 7; LPL, lipoprotein lipase; MMP-2, matrix metalloproteinase 2; NLR, neutrophil-to-lymphocyte ratio; NT-proBNP, N-terminal prohormone of brain natriuretic peptide; RAGE, receptor for advanced glycation endproducts; RET, receptor tyrosine kinase RET; ST2, soluble suppression of tumorigenicity 2 or interleukin 1 receptor, type I (IL1R1); TIMP1, tissue inhibitor of metalloproteinase 1; TIMP2, tissue inhibitor of metalloproteinase 2; TNF-α, tumor necrosis factor alpha; TRAIL, tumor necrosis factor-related apoptosis-inducing ligand; vWF, von Willebrand factor.

The selection of candidate biomarkers was done according to the following criteria: (1) Biomarkers addressing an unmet medical need in relation to pulmonary arterial hypertension; (2) The biomarker(s) providing new or additional information to aid in clinical decisions; (3) analytical validation (accessible, accurate, validated, and reproducible methods used); (4) existent regulatory approvals or used in other conditions/diseases; and (5) clinical validation (data supporting the relationship between the biomarkers and the outcome of interest). Other parameters not considered in this table include cost-efficiency and availability.

a One or several of the biomarkers are already approved/established in routine practice of other conditions/diseases but not in pulmonary arterial hypertension.

Last, increased collaborations and shared biobanks in recent years have prompted the identification of a selection of promising biomarker candidates in PAH- diagnosis, prognosis, monitoring, and susceptibility/risk prediction. In conclusion, for a biomarker to be clinically utilized, the following should be fulfilled: (1) accessible, accurate, and reproducible methods for measurement at a reasonable cost, (2) the biomarker must be validated and provide new or additional information, and (3) aid in clinical decision-making.^112^

## Author contributions

Salaheldin Ahmed: conceptualization, methodology, software, investigation, writing – original draft, formal analysis, visualization. Abdulla Ahmed: conceptualization, methodology, visualization, investigation, writing – review and editing. Göran Rådegran: conceptualization, investigation, resources, writing – review and editing, supervision, project administration. All authors are accountable for all aspects of the work and approve the final version to be published.

## Funding

The work was supported by funding from “Avtal om Läkarutbildning och Forskning” (ALF), Skåne University Hospital’s foundations and donations, Go Rad Care AB, and the Department of Education and Research of Helsingborg’s Hospital. The funding organizations played no role in the collection, analysis or interpretation of the data and had no right to restrict the publishing of the manuscript.

## Disclosure statement

The authors declare the following financial interests/personal relationships which may be considered as potential competing interests: Dr Salaheldin Ahmed and Dr Abdulla Ahmed report financial support was provided by Go Rad Care AB and the Department of Education and Research of Helsingborg’s Hospital. Dr Göran Rådegran reports financial support was provided by Avtal om läkarutbildning och forskning (ALF), and Skåne University Hospital’s foundations and donations. Dr Salaheldin Ahmed and Dr Abdulla Ahmed report a relationship with Janssen-Cilag AB and Nortic Infucare that includes consulting or advisory. Dr Göran Rådegran reports a personal relationship with Actelion Pharmaceuticals Sweden AB, Bayer Health Care, GlaxoSmithKline, Janssen-Cilag AB, and Nordic Infucare that includes consulting or advisory. Dr Rådegran is, and has been primary-, or co-, investigator in clinical PAH trials for Acceleron, Actelion Pharmaceuticals Sweden AB, Bayer, GlaxoSmithKline, Janssen-Cilag AB, MSD, Pfizer, and United Therapeutics, and in clinical heart transplantation immunosuppression trials for Novartis.

## Appendix

**Methods**

Entry terms:

1. PubMed (Medline, PMC, NCBI Bookshelf)
  “(("Pulmonary Arterial Hypertension"[Mesh]) OR ("pulmonary arterial hypertension") OR ("pulmonary artery hypertension") OR ("Vascular Remodeling"[Mesh] AND "Pulmonary Arterial Hypertension"[Mesh])) AND ("Biomarkers"[Mesh] OR" Proteome"[Mesh]) NOT ("Review" [Publication Type]) AND ("Journal Article" [Publication Type]) NOT ("Overall" [Publication Type]) AND ((humans[Filter]) AND (2012/1/1:2023/9/15[pdat]) AND (english[Filter]))”.
2. Embase
  “”('pulmonary arter* hypertension' OR (('vascular remodeling' OR 'vascular remodeling'/exp) AND 'pulmonary arter* hypertension')) AND ('biological marker'/exp OR proteome OR 'plasma biomarkers') AND [english]/lim AND [article]/lim AND [humans]/lim AND [2012–2023]/py””.
